# Supplementary material for: Pathways to optimising antibiotic use in rural China: identifying key determinants in community and clinical settings, a mixed methods study protocol
Source: BMJ Open. 2019 Aug 10;9(8):e027819. doi: 10.1136/bmjopen-2018-027819 (PMC6701592; doi:10.1136/bmjopen-2018-027819)
Supplement: Supplementary data [file bmjopen-2018-027819supp001.pdf]

Appendix

Study dates: June 2016 to November 2019

Table 1 Data collection

Component	Start Date Pilot	End Date Pilot	Start Date Site 1	End Date Site 1	Start Date Site 2	End Date Site 2	Start Date Site 3	End Date Site 3
Microbiology	Month 11	Month 12	Month 16	Month 20	Month 21	Month 24	Month 28	Month 31
Exit Interviews	Month 11	Month 12	Month 16	Month 20	Month 21	Month 24	Month 28	Month 31
Patient In-Depth Interviews	Month 12	Month 13	Month 19	Month 20	Not required	Not required	Not required	Not required
Recruitment Proforma [Observations]	Month 10	Month 12	Month 16	Month 20	Month 21	Month 24	Month 28	Month 31
Pharmacy Observations & Exit Interviews	Month 12	Month 13	Month 19	Month 20	Month 23	Month 24	Not required	Not required
Record Review	Month 13	Ongoing review	Month 25	Ongoing review	Month 25	Ongoing review	Not required	Not required

Table 2 Organisms to be analysed related to specimen and antibiotics tested

specimen	isolate	agents tested
urine	*E.coli*<br>*Klebsiella spp*<br>*Proteus spp*	amoxicillin, co-amoxiclav, trimethoprim/ co-trimoxazole, ciprofloxacin, gentamicin, cefotaxime/ceftriaxone
throat swabs	*B.haemolytic streptococci*<br>*Haemophilus spp*	penicillin, macrolide,<br>Blactamase production,<br>quinolone
	Moraxella catarrhalis<br>*S.aureus*	Blactamase production, cefoxitin/oxacillin, macrolide
	S.pneumoniae<br>*Enterobacteriales*<br>*N.meningitidis*	penicillin, macrolide, amoxicillin, co-amoxiclav, trimethoprim/ co-trimoxazole, ciprofloxacin, gentamicin, cefotaxime/ceftriaxone
sputum	*S.pneumoniae*<br>*Haemophilus spp*<br>*Moraxella catarrhalis*<br>*S.aureus*<br>*Enterobacteriales*<br>*B.haemolytic streps*<br>*N.meningitidis*	agents to test as for throat swabs
