# Supplementary material for: Pathways to optimising antibiotic use in rural China: identifying key determinants in community and clinical settings, a mixed methods study protocol
Source: BMJ Open. 2019 Aug 10;9(8):e027819. doi: 10.1136/bmjopen-2018-027819 (PMC6701592; doi:10.1136/bmjopen-2018-027819)
Supplement: Author's manuscript [file bmjopen-2018-027819.draft_revisions.pdf]

BMJ Open

BMJ Open is committed to open peer review. As part of this commitment we make the peer review history of every article we publish publicly available.

When an article is published we post the peer reviewers' comments and the authors' responses online. We also post the versions of the paper that were used during peer review. These are the versions that the peer review comments apply to.

The versions of the paper that follow are the versions that were submitted during the peer review process. They are not the versions of record or the final published versions. They should not be cited or distributed as the published version of this manuscript.

BMJ Open is an open access journal and the full, final, typeset and author-corrected version of record of the manuscript is available on our site with no access controls, subscription charges or pay-per-view fees (http://bmjopen.bmj.com).

If you have any questions on BMJ Open's open peer review process please email info.bmjopen@bmj.com

BMJ Open

Pathways to optimising antibiotic use in Anhui: Identifying key determinants in community and clinical settings, a mixed methods study protocol.

Journal:	*BMJ Open*
Manuscript ID	bmjopen-2018-027819
Article Type:	Protocol
Date Submitted by the Author:	12-Nov-2018
Complete List of Authors:	Zhao, Linhai; Anhui Medical University, School of Health Services Management<br>Kwiatkowska, Rachel; Public Health England, Field Service, National Infection Service; University of Bristol Medical School, School of Population Health Sciences, NIHR Health Protection Research Unit in Evaluation of Interventions<br>Chai, Jing; Anhui Medical University, School of Health Service Management<br>Cabral, Christie; University of Bristol Medical School, Centre for Academic Primary Care<br>Chen, Meixuan; University of Bristol Medical School, School of Population Health Sciences<br>Bowker, Karen; North Bristol NHS Trust, Severn Pathology<br>Coope, Caroline; Public Health England, Field Service, National Infection Service; University of Bristol Medical School, School of Population Health Sciences, NIHR Health Protection Research Unit in Evaluation of Interventions<br>Shen, Jilu; First Affiliated Hospital of Anhui Medical University<br>Shen, XingRong; Anhui Medical University, School of Health Services Management<br>Cheng, Jing; Anhui Medical University, School of Health Services Management<br>Feng, Rui; Anhui Medical University, Library Department of Literature Retrieval and Analysis<br>Kadetz, Paul; Drew University; Xi'an Jiaotong-Liverpool University<br>MacGowan, Alasdair; North Bristol NHS Trust, Severn Pathology<br>Oliver, Isabel; University of Bristol School of Population Health Sciences, NIHR Health Protection Research Unit in Evaluation of Interventions; Public Health England, Field Service, National Infection Service<br>Hickman, M; University of Bristol Medical School, School of Population Health Sciences; University of Bristol Medical School, School of Population Health Sciences, NIHR Health Protection Research Unit in Evaluation of Interventions<br>Wang, DeBin; Anhui Medical University, School of Health Services Management<br>Lambert, Helen; University of Bristol Medical School, School of Population Health Sciences
Keywords:	Antimicrobial Resistance, Antibiotics, Mixed methods, China, Outpatient,

For peer review only - http://bmjopen.bmj.com/site/about/guidelines.xhtml

	Medical Records

SCHOLARONE™
Manuscripts

Pathways to optimising antibiotic use in Anhui: identifying key determinants in community and clinical settings, a mixed methods study protocol.

Linhai Zhao[1]*; Rachel Kwiatkowska[2,3]*; Jing Chai[1]; Christie Cabral[4]; Meixuan Chen[5]; Karen Bowker[6]; Caroline Coope[2,3]; Jilu Shen[7]; Xinrong Shen[1]; Jing Chen[1]; Rui Feng[8]; Paul Kadetz[9-11]; Alasdair MacGowan[6]; Isabel Oliver[2,3]; Matthew Hickman[2,5]; Debin Wang[1]; Helen Lambert[5]

1. School of Health Service Management, Anhui Medical University, 81 Meishan Road, Hefei, 230032, China.
2. NIHR Health Protection Research Unit (HPRU) in Evaluation of Interventions, Bristol Medical School, Population Health Sciences, University of Bristol, Oakfield House, Oakfield Grove, Bristol, BS8 2BN, UK
3. Field Service, National Infection Service, Public Health England, 3rd floor, 2 Rivergate, Bristol, UK BS1 6EH, UK
4. Centre for Academic Primary Care, Bristol Medical School, University of Bristol, Canynge Hall, 39 Whatley Road Bristol BS8 2PS, UK
5. Department of Population Health Sciences, Bristol Medical School, Bristol Medical School, Canynge Hall, 39 Whatley Road, Bristol BS8 2PS, UK
6. Severn Pathology, North Bristol NHS Trust, Pathology Building, Southmead Hospital, Southmead Road, Westbury-On-Trym, Bristol BS10 5NB, UK
7. First Affiliated Hospital of Anhui Medical University, 218 Jixi Rd, Shushan Qu, Hefei Shi, Anhui Sheng, China, 230022
8. Library of Anhui Medical University, 81 Meishan Road, Hefei, 230032, China
9. Drew University, 36 Madison Ave, Madison, NJ 07940, USA
10. Xi'an Jiaotong Liverpool University, Wuzhong, Suzhou, China, 215123
11. China Centre for Health and Humanity, University College London, Gower Street, WC1E 6BT, UK

*LZ and RK contributed equally to this manuscript.

Correspondence to: Dr R M Kwiatkowska, NIHR Health Protection Research Unit (HPRU) in Evaluation of Interventions, Bristol Medical School, Population Health Sciences, University of Bristol, Oakfield House, Oakfield Grove, Bristol, BS8 2BN, UK. Tel. +44 (0)117 3310177 Email rachel.kwiatkowska@bristol.ac.uk

Keywords: Antimicrobial Resistance; Antibiotics; Mixed Methods; China; Medical Records; Outpatients

Word count: 4115

For peer review only

Abstract

Introduction: This study aims to investigate patterns of antibiotic treatment-seeking, describe current levels of and drivers for antibiotic use for common infections (respiratory tract and urinary tract infections), and test the feasibility of determining the prevalence and epidemiology of antimicrobial resistance (AMR) in rural areas of Anhui Province, in order to identify potential interventions to promote antibiotic stewardship and reduce the burden of AMR in China.

Methods and analysis: We will conduct direct observations, structured and semi-structured interviews in retail pharmacies, village clinics and township health centres to investigate treatment-seeking and antibiotic use. Clinical isolates from 1550 sputum, throat swab and urine samples taken from consenting patients at village and township health centers will be analysed to identify bacterial pathogens and ascertain antibiotic susceptibilities. Healthcare records will be surveyed for a sub-sample of those recruited to the study, to assess their completeness and accuracy.

Ethics and dissemination: The full research protocol has been reviewed and approved by the Biomedical Ethics Committee of Anhui Medical University (reference number: 20170271). Participation of patients and doctors are voluntary and written informed consent is sought from all participants. Findings from the study will be disseminated through academic routes including peer-reviewed publications and conference presentations, via tailored research summaries for health professionals, health service managers and policymakers, and through an end of project impact workshop with local and regional stakeholders to identify key messages and priorities for action.

Strengths and limitations of this study:

- This innovative cross-disciplinary study will combine qualitative, microbiological and epidemiological methodologies to investigate drivers of AMR in rural China.
- It will systematically document key drivers of, and patient pathways leading to, antibiotic use and establish the feasibility of microbiological testing and epidemiological monitoring for AMR and antibiotic use at front-line medical settings in rural China.
- The mixed methods approach will provide a comprehensive picture of factors influencing prescribing and sampling practices, so that bias arising from any individual dataset can be accounted for in the analysis and interpretation of results strengthened through triangulation.
- The study area, though selected to be representative of rural populations in China, only includes three rural counties of 105 counties in Anhui province. As such, results may have limited generalisability.
- Nonetheless, the study will identify modifiable influences on current treatment-seeking pathways leading to antibiotic use and determine the feasibility of measuring and monitoring AMR in rural populations; these findings will inform efforts to reduce inappropriate prescribing and improve antimicrobial stewardship.

For peer review only

Introduction

The problem of drug-resistant infections is widely acknowledged to be of global public health concern, with non-essential use of antibiotics in humans a major contributor.[1] There is substantial evidence that antibiotics are overused in the Chinese health care system.[2-8] A range of possible reasons for unnecessary use of antibiotics have been identified, including 'demand side' issues such as cultural expectations and social norms leading patients to expect antibiotic treatment, and 'supply side' issues such as perverse financial incentives for prescribing antibiotics, systemic pressures on healthcare providers and professional norms which lead to overprescribing as a risk-reduction strategy. Of these, service providers may play a dominant role.

Health professionals in the UK and China often claim that antibiotic overuse is driven by patient demand and link this, in turn, to lack of public understanding of antibiotic effectiveness and low public awareness. [9] Studies in both the UK and China suggest however that 'patient demand' may actually be a misperception on the part of health care providers, or a rationalisation of their own antibiotic prescribing practices.[10-13] A study of self-medication in Hefei City, Anhui province, found an association between higher maternal education, medical insurance cover and non-prescribed use of antibiotics for children's illnesses.[14] A UK study of prescribing in primary care showed doctors' perceptions of patient pressure to be a consistently stronger predictor of prescribing than patients' actual preferences.[15] On the other hand, it is not uncommon for doctors in both the UK and China to prescribe antibiotics 'just in case', due to a desire to reduce medical, legal and reputational risk in the face of clinical uncertainty.[16 17]

A cluster-randomized survey was conducted during the development of this protocol and 2600 rural residents in 12 counties of Anhui Province were asked about self-reported health service-seeking and antibiotic consumption. Almost three quarters (72.7%) of respondents reported that they would do as told if their doctors let them leave an appointment without prescribing any medicine, and only 14.3% had ever asked their doctors to prescribe specific drugs.[18] Results also suggested that knowledge about antibiotics (which pathogens they can be used to treat, and what the potential risks are of using them) was associated with increased purchasing of over-the-counter pharmaceuticals and with re-use of leftover pharmaceuticals from family or friends.

There is evidence from China that health systems, especially remuneration mechanisms, may be important drivers of unnecessary antibiotic use. [19] Patients are used to paying only for medicines they are given and medicine forms an important source of revenue for all care providers. Historically, remuneration mechanisms have provided a perverse financial incentive for health care practitioners to overprescribe antibiotics, particularly at lower levels of the health system. [20] Recent government health care reforms, together with policies such as the Special Antimicrobial Use Rectification program, have been associated with reductions in antibiotic prescribing at county and higher level public hospitals.[3] However, current policies aim to reduce overall use (for example by capping the overall proportion of antibiotic prescriptions at hospitals), but do not necessarily ensure equitable access to essential treatment;

For peer review only

restricting overall availability of antibiotics may exacerbate health inequities through its negative effects on access to effective treatment among the poor. [21]

Current policies and interventions mainly focus on antibiotic prescribing, but in many settings non-prescribed antibiotics are widely consumed. In China, antibiotics are freely available over-the-counter without prescription, but little is known for either urban or rural settings about what prompts people to seek treatment at different health facilities or the role of the informal sector (including self-medication, retail pharmacies, traditional Chinese medicine practitioners, unqualified bio-medicine practitioners and home care) in the management of infections.

Microbiological facilities are not available at most front-line health care settings. Most research on AMR in China has therefore drawn on patient data collected in urban teaching hospitals. Because patients can choose to attend facilities at any level of the Chinese health care system and care pathways do not follow a predictable pattern of upwards referral, the extent to which antibiotic prescribing in urban hospitals drives the spread of antibiotic resistance in the wider population is currently unknown. The often asserted high burden of antimicrobial resistance in China may be misleading since current assumptions are based on potential pathogens isolated in selected microbiology laboratories enrolled in the national surveillance system and it is uncertain if this reflects the incidence of resistance in non-hospitalised patients with mild to moderate infection receiving antibiotics, most of whom do not have samples sent to the laboratory.[22 23] UK data indicates that 1:35 patients presenting to their GPs with acute cough have microbiological sampling and perhaps 1:5 with UTI.[24] This data indicates that resistance rates to key antibiotics based on biased laboratory data may be over-emphasised.[25 26]

There is a need for comprehensive and systematic assessment of prescribing and purchasing practices in the context of China's unique health systems and policies, to identify potential targets for interventions to optimise prescribing and consumption. There are also crucial gaps in evidence regarding antibiotic resistance and its determinants in rural communities and health facilities at village and township levels in China and it is important to investigate the possibility of introducing routine monitoring of prevalence and epidemiology of AMR in these settings.

Study aims and objectives

This study aims to investigate levels of antibiotic prescribing and the burden of AMR in rural areas in China and identify key drivers of antibiotic use for common infections in the community, in order to identify potential interventions to promote antibiotic stewardship and reduce the burden of AMR.

The study objectives are summarised in table 1.

For peer review only

Table 1 Study objectives

**1**	Assess feasibility of obtaining community-based samples, isolating common pathogens and determining their antibiotic susceptibilities for presentations of Urinary Tract Infections (UTIs) and Respiratory Tract Infections (RTIs);
**2**	Estimate prevalence of AMR in non-hospitalised patients and test the hypothesis that community prevalence of AMR is lower than that based on analyses of samples from large urban hospitals with a higher proportion of inpatients;
**3**	Describe patient clinical record systems and assess the reliability of information from electronic patient records for monitoring antimicrobial usage and AMR;
**4**	Analyse microbiological findings in relation to patient-reported symptoms, clinical diagnoses and antibiotics prescribed in clinical facilities;
**5**	Investigate what types and sources of health care rural residents of Anhui Province seek for common respiratory tract infections from all forms and sources of health care (including informal, Traditional Chinese Medicine);
**6**	Understand what drives antibiotic provision (both prescribed and purchased without prescription) and consumption for selected conditions at lower levels of the formal and informal health care system; and
**7**	Assess what types of and targets for intervention are likely to be most effective for optimizing antibiotic use.

Methods and analysis

Study design

This is a mixed-method study comprising two main components: a microbiological feasibility study and clinical record review (objectives 1-4), and a qualitative study of antimicrobial prescribing and purchasing practices (objectives 5-7).

Setting

This study is being implemented in three rural residential areas in Anhui Province, including one village clinic, one township health centre and all local pharmacies (4-8) within each area. One of the three areas will be used to pilot the study. Anhui Province is located in northeast China and has a population of 68.6 million of whom 57% live in rural areas. Per capita GDP and income in Anhui rank in the middle (14th) among all provinces in the nation. Its social, cultural and economic background is representative of over 80% of the population in China. There are 968 hospitals, 1,941 community & 1,398 township health centres, and 15,288 village clinics in the province (Annual Health Anhui Statistics 2014). As in most other provinces, there is no strict referral system in Anhui and patients may choose from any level and kind of caregiver, though medical insurance systems enact a 10 to 20% decreasing reimbursement ratio for direct medical expenditures incurred at primary care settings upward. In terms of first time medical care, 62.9% is at village or community clinics, 17.3%, at township or community health centres and 13.6%, at county level hospitals (Annual Anhui Health Statistics 2014).

For peer review only

Participants

The microbiological sub-study aims to recruit a total of 1,550 patients (1,000 with exacerbation of COPD and 555 with UTI). According to our calculations, this should provide 200 *E coli* isolates and 100 *Strep pneumonieae* isolates which should allow us to estimate antibiotic susceptibilities with precision, on the assumption that:

- 40% of UTI samples will yield a pathogen
- 25% of COPD samples will yield a pathogen
- 90% of pathogens isolated from urine will be *E.coli*
- 40% of pathogens isolated from sputum will be *S.pneumoniae*
- In *E.coli*, resistance to nitrofurantoin, fosfomycin and co-amoxiclav will be <5% and resistance to cephalosporins and fluoroquinolones 50-60% [27]
- In *S.pneumoniae*, penicillin resistance will be 12.5% and erythromycin resistance 90% [28]

Inclusion criteria of patients are male or female who are: a) 18 years or older and able to give consent to participate in the microbiological study, exit survey and/or follow-up interviews; b) presenting to the recruitment site for his/her current illness for the first time during the study period; and c) diagnosed by the attending doctor as having one or more of the following: urinary tract infection, exacerbation of chronic obstructive pulmonary disease, upper respiratory tract infection with productive cough, and sore throat. These patients will be selected via "consecutive sampling" in which, when a start date has been determined, the recruitment continues daily (7 days a week) thereafter, between 8am-5pm or 9am-6pm on alternate days, until the target numbers have been reached. All incoming patients to the site village clinics and township health centres who meet the inclusion criteria during any study day are invited to participate. This is a pragmatic approach to sampling since patient record systems do not allow the flexibility to carry out random sampling, and ongoing recruitment will ensure the most efficient use of staff and resources in this setting.

Qualitative study participants will include: a) patients visiting the selected village clinics and township health centers during the study period; b) customers visiting the selected retail pharmacies during the study period; c) doctors within the clinics or health centers for treatment of RTIs/UTIs; d) salespersons working within the selected retail pharmacies.

Data collection

Specimen collection and microbiological testing (objectives 1-2, 4)

The study will collect sputum, throat swab and urine specimens for pathogen identification and susceptibility testing. Sputum will be collected from RTI patients with productive cough (e.g., exacerbation of Chronic Obstructive Pulmonary Disease (COPD); throat swabs from patients with sore throat; and urine from patients with UTIs. Specimen collection will be performed by the attending doctor and placed into a sterilised tube according to a standard operation protocol. The specimen tubes will be put immediately into a refrigerator and preserved at below –4℃ temporarily prior to being shipped to the Central Laboratory of Anhui Medical University (AMU). At about 12:00 pm and 5:30 pm each day, the samples in the refrigerator will be transferred to a portable code case filled with ice bags which is then sealed and handed over to a contracted bus company. The company is held responsible for shipping the specimens within

For peer review only - http://bmjopen.bmj.com/site/about/guidelines.xhtml

a set time limit (within 4 hours).

Upon arrival of any batch of specimens at AMU central lab, a designated technician will perform standard specimen assessment and pre-treatment and then bacteria inoculation, identification, and susceptibility tests. Sputum and throat swab specimens will be inoculated on four plates (a blood agar plate, a MacConkey agar plate, a chocolate agar plate and a chrome agar plate); and urine specimens inoculated on two plates (a blood agar plate and a MacConkey agar plate). The inoculated blood agar and chocolate agar plates will be cultured and incubated in r 5% CO_2 and 35^0C; the MacConkey agar and the chrome agar plates, incubated in air at 35^0C. The incubation time will be 18-24 hours plus an additional 24 hours if no bacteria are observed for the first period. Bacteria identification will use automated methods i.e., the MicroscanWalkaway-96 System with PC33 (for gram positive bacteria) or NC50 (for gram negative bacteria) kits, and also according to different biochemical reactions as follows: a) E.coli chrome agar/ VITEK2 or MALDI-TOF; b) S.pneumoniae optochin/ biochemistry; c) H.influenzae X+V factors or MALDI-TOF; and d) M.catarrhalis MALDI-TOF. For the bacteria susceptibilities, automated methods will be used (VITEK2 and MicroScan Walk Away 96 plus) along with disk diffusion and agar dilution methods (according to CLSI). Antimicrobial susceptibility data on the key pathogens identified in community sampling will be extracted from the existing diagnostic laboratory databases for comparison with isolates from village clinics and county hospitals.

Electronic Record Review (objectives 3, 4)

Medical record-keeping systems in Anhui province will be described in detail using information from clinic observations and consultations with clinical personnel and medical centre directors. Outpatient electronic care systems will be interrogated two weeks after patient recruitment and compared with notes from clinic observations to assess completeness and accuracy of electronic care records. A one year sample of all electronic care records from the participating facilities will then be drawn to investigate patterns and rates of antibiotic prescribing in relation to recorded diagnosis, clinical indication and laboratory results.

Structured exit surveys (objective 5, 6)

Exit surveys will be carried out with patients presenting with symptoms of sore throat, respiratory tract infection (RTI) or urinary tract infection (UTI) following either their consultation at a health care facility or their visit to a retail pharmacy. Those who consent to being recruited will complete the survey prior to leaving the facility. Using a brief semi-structured questionnaire developed from open-ended interview responses in the study's pilot phase, the survey will solicit details of symptoms, perception of the problem (lay diagnosis), professional and/or self-treatment taken before the visit, whether the diagnosis and treatment received (including prescribed antibiotics) is as expected, as well as views on specimen collection and microbiological testing in patients attending the health care facility.

Semi-structured observations (objectives 3, 5, 6)

These will occur at all the participating clinics, health centers and retail pharmacies. At clinics

For peer review only - http://bmjopen.bmj.com/site/about/guidelines.xhtml

and health centers, the observation focuses on daily operational routine including test ordering, prescribing, patient recall and other standard procedures using a pre-designed worksheet, together with observational notes on clinical encounters. At retail pharmacies, the observation uses a similar worksheet and documents daily encounters between customers and shop assistants including the health problems presented, and medicines and information asked for by the customers and/or suggestions made, and medicines and information given by the assistants.

In-depth interviews (objectives 5, 6)

These will involve a sub-sample of the patients (around 60 in total based on previous study experience of numbers needed to reach data saturation) and customers (around 60) who have completed the exit survey and all the related doctors and salespersons who have served the participating patients or costumers. Interviews with individual patients and customers will be undertaken respectively at their homes and at the pharmacy one-two weeks after their initial visit; interviews with individual doctors and salespersons will take place any time during the study period when they are free. Topic guides refined during the pilot phase of the study will be used to focus patient interviews on the illness episode for which treatment was sought and any previous action taken to manage the illness, while interviews with doctors focus on the following main topics: professional experience and expertise, normal practice, prescribing antibiotics, patient influences on antibiotic prescribing and external influences on antibiotic prescribing.

A sub-sample of interviewees will take part in pile sorts, a qualitative method in which informants are provided with a set of index cards each showing a pertinent term and asked to group the cards according to their perceived appropriate clustering of terms. Terms will be antibiotics-related and derived from earlier exit surveys, observations and in-depth interviews.[29 30] This method provides another means to distinguish how informants understand antibiotics and their use.

Data analysis

Quantitative analysis (objectives 1-4)

Data from the microbiological testing, review of clinical records and exit surveys will be analysed quantitatively to investigate prevalence of infection and rates of resistance, relationships between infection/ resistance rates and patient characteristics, and feasibility of routine microbiological testing in rural clinics. The feasibility of obtaining community-based samples of common pathogens and determining their antibiotic sensitivities **(objective 1)** will be examined by: a) ratios of patients who have provided specimens versus those who have refused and their reasons for doing so; b) percentages of specimens assessed as being properly collected, preserved and shipped by designated microbiological technicians; c) ratios of positive versus negative attitudes toward giving specimens among the exit interviewees and views of the participating doctors. Prevalence of pathogens and antimicrobial resistance **(objective 2)** will be assessed by: a) estimating percentages of pathogen positive specimens in terms of all and specific bacteria by diagnosis, type of specimen, patient reported symptoms, previous

For peer review only

treatment history, and socio-demographics; b) calculating percentages of resistant pathogens by type of bacteria and antibiotics; and c) comparing, using chi-square-test, these pathogen and resistance rates in non-hospitalised patients with those from analyses of data from large urban hospitals. We will produce a descriptive analysis of patient record systems and electronic record completeness and accuracy **(objective 3)** and use logistic regression to explore relationships between pathogen types and susceptibilities and patient characteristics such as presenting symptoms, clinical diagnosis, prior use of antibiotics and demographics **(objective 4).**

Qualitative analysis (objectives 5-7)

Analysis of the qualitative data (in-depth interviews, observations) will focus on identifying characteristic patterns in treatment-seeking pathways to antibiotic use among patients and antibiotic purchasers (**objective 5**) and on identifying key social, cultural, health systems and behavioural determinants of both antibiotic prescribing and provision among health care providers and of antibiotic use among residents (**objective 6**). Thematic and framework analysis will be undertaken for each of the four qualitative datasets using a similar approach. Following translation of a subset of interviews from each data set, members of the research team will independently free code a minimum of three interview transcripts in Mandarin or English and then meet to compare and finalise a code list. Further interviews will be coded thematically in batches in the software package NVivo 12 using the agreed codes until all data have been coded. Each interviewer will also summarise each interview transcript by entering a summary of the coded data in a Framework using Excel. Pile sorts will be analysed using Johnson's Hierarchical Cluster analysis, providing a two-dimensional concept mapping of the terms that are most frequently grouped together.

The occurrence of resulting themes will also be used to produce theme frequency maps and theme networks via NVivo or UCINET for different groups of participants and different topics.

Analysis of antibiotics-related pathways comprises identifying both a complete set of paths for common RTIs/UTIs and factors affecting patient trajectories among these pathways. Anticipated path sets to be produced include a general path set applicable for both RTIs and UTIs and an RTI and UTI-specific path set respectively. Similarly, factor sets of interest in this study comprise combinations of factors that affect selection between: a) doing nothing versus taking action; b) self-care versus professional treatment; c) western medicine versus traditional Chinese medicine; d) antibiotics versus non-antibiotics therapy.

Analytic synthesis

Development of interventions for optimizing antibiotics use (**objective 7**) will build upon findings from both quantitative and qualitative analyses. It will also analyse the pathways and determinants identified from this study in relation to the Behaviour Change Wheel, a theoretical framework developed from 19 frameworks of behaviour change identified in a systematic literature review.[31] The COM-B ('capability', 'opportunity', 'motivation' and 'behaviour') model, which recognizes behaviour as part of an interacting system, will be used to identify key modifiable influences leading to the use of antibiotics and subsequently, inform

For peer review only - http://bmjopen.bmj.com/site/about/guidelines.xhtml

recommendations for interventions to optimise antibiotic use.

Patient and Public Involvement

Development of the research question was informed by patients' experiences and preferences as documented in a previous study.[18] The results will be disseminated to study participants and other stakeholders through a local dissemination workshop to be held at the end of this study and through short summaries of research findings.

Ethics and dissemination

This protocol is rooted in a multidisciplinary collaboration between institutions in the UK and China. Investigators will not only benefit from exchange of academic knowledge and methodologies but will gain a situational awareness which extends beyond their own area of expertise. This will lead to greater depth of insight into the scope and interrelatedness of influencing factors and allow the team to analyse and interpret study results with full awareness of the complexity of drivers of AMR and antibiotic use in rural Anhui province. The research will provide academics with a documented case study of how to conduct large scale research involving a multi-disciplinary team across two countries, as well as a 'worked example' of research capacity-building through academic collaboration for the benefit of a middle-income country. Regular workshops will allow the China and UK research teams to exchange ideas and insights throughout the duration of the study. Lessons from this study will be shared with the wider academic community through peer-reviewed publications and conference presentations.

This innovative study will provide the first systematic documentation of routes and means to antibiotic use by residents at lower ends of the healthcare system (township and village clinics) in China and assess the feasibility of routine microbiological testing and analysis of antimicrobial sensitivities in the context of information from clinic healthcare records.

Findings from the study will be summarised for the benefit of public health policymakers, health professionals and health service managers in China and elsewhere. A final workshop will be held for the purpose of presenting the evidence generated by this study, and working with local and regional stakeholders to translate this evidence into public health action.

The Biomedical Ethics Committee of Anhui Medical University has provided full ethical approval for this study (reference number: 20170271). Participation in the study is fully voluntary for patients, doctors and pharmacy staff, all of whom will give written informed consent to participate.

Authors' contributions:

RK, LZ and JChai wrote the manuscript with supervision and contributions from project leads AMacG (microbiology component), IO and MH (record review component), PK (social sciences component) and Principal Investigators HL (UK) and DW (China).

CCo and PK contributed to developing the initial study collaboration and to revision of the

For peer review only

manuscript. RF, JCheng, JChai, MC and CCa contributed to qualitative research tool design including methods for data collection and analysis. KB and JS developed methods for microbiology sample collection and laboratory analysis. IO, RK and XS developed methods for record review data extraction and analysis. XS also designed a central database for storing data from all components of the study. HL, AMacG, IO, MH, PK and DW conceived and designed the original study. All authors reviewed the manuscript.

Acknowledgements

The authors gratefully acknowledge the contributions of Professor Susan Michie and Dr. Annegret Schneider (University College London), Professor Alastair Hay (University of Bristol), and Professor Anthony Kessel (Public Health England) in the conception of this study.

Competing interests statement: None

Funding statement: This work was supported by the Newton Fund (UK Research and Innovation (UKRI) and the National Natural Science Foundation of China (NSFC)) under the UK-China AMR Partnership Initiative, grant number MR/P00756/1.

RK, CCo, MH and IO all acknowledge support from the NIHR Health Protection Research Unit in Evaluation of Interventions at the University of Bristol.

References

1. WHO. Global Action Plan on Antimicrobial Resistance: World Health Organisation, 2015.
2. Li Y, Xu J, Wang F, et al. Overprescribing In China, Driven By Financial Incentives, Results In Very High Use Of Antibiotics, Injections, And Corticosteroids. *Health Affairs* 2012;31:1075-82. doi: 10.1377/hlthaff.2010.0965
3. Sun Q, Dyar OJ, Zhao L, et al. Overuse of antibiotics for the common cold – attitudes and behaviors among doctors in rural areas of Shandong Province, China. *BMC Pharmacology and Toxicology* 2015;16:6. doi: 10.1186/s40360-015-0009-x
4. Xiao Y, Li L. Legislation of clinical antibiotic use in China. *The Lancet Infectious Diseases* 2013;13(3):189-91. doi: 10.1016/s1473-3099(13)70011-2
5. Xiao Y, Zhao K, Bishai DM, et al. Essential drugs policy in three rural counties in China: what does a complexity lens add? *Soc Sci Med* 2013;93:220-8. doi: 10.1016/j.socscimed.2012.09.034 [published Online First: 2012/10/30]
6. Yang C, Yang S, Yang L, et al. The impact of the national essential medicines policy on rational use of antibiotics in China. *Chinese Journal of Health Service Management* 2014;308:109-12.
7. Yin X, Song F, Gong Y, et al. A systematic review of antibiotic utilization in China. *Journal of Antimicrobial Chemotherapy* 2013;68:2445-52. doi: 10.1093/jac/dkt223
8. Zhang R, Eggleston K, Rotimi V, et al. Antibiotic resistance as a global threat: evidence from China, Kuwait and the United States. *Global Health* 2006;2:6. doi: 10.1186/1744-8603-2-6 [published Online First: 2006/04/11]

For peer review only

9. Cole A. GPs feel pressurised to prescribe unnecessary antibiotics, survey finds. *BMJ (Clinical research ed)* 2014;349:g5238. doi: 10.1136/BMJ.G5238

10. Brookes-Howell L, Wood F, Verheij T, et al. Trust, openness and continuity of care influence acceptance of antibiotics for children with respiratory tract infections: a four country qualitative study. *Family Practice* 2014;31:102-10. doi: 10.1093/fampra/cmt052

11. Cabral C, Horwood J, Hay AD, et al. How communication affects prescription decisions in consultations for acute illness in children: a systematic review and meta-ethnography. *BMC Family Practice* 2014;15:63. doi: 10.1186/1471-2296-15-63

12. Lucas PJ, Cabral C, Hay AD, et al. A systematic review of parent and clinician views and perceptions that influence prescribing decisions in relation to acute childhood infections in primary care. *Scandinavian Journal of Primary Health Care* 2015;33:11-20. doi: 10.3109/02813432.2015.1001942

13. McCullough AR, Rathbone J, Parekh S, et al. Not in my backyard: a systematic review of clinicians' knowledge and beliefs about antibiotic resistance. *J Antimicrob Chemother* 2015;70(9):2465-73. doi: 10.1093/jac/dkv164 [published Online First: 2015/06/21]

14. Bi P, Tong S, Parton KA. Family self-medication and antibiotics abuse for children and juveniles in a Chinese city. *Social science & medicine (1982)* 2000;50:1445-50.

15. Little P, Dorward M, Warner G, et al. Importance of patient pressure and perceived pressure and perceived medical need for investigations, referral, and prescribing in primary care: nested observational study. *BMJ* 2004;328:444-0. doi: 10.1136/bmj.38013.644086.7C

16. He AJ. The doctor–patient relationship, defensive medicine and overprescription in Chinese public hospitals: Evidence from a cross-sectional survey in Shenzhen city. *Social Science & Medicine* 2014;123:64-71. doi: https://doi.org/10.1016/j.socscimed.2014.10.055

17. Cabral C, Lucas PJ, Ingram J, et al. "It's safer to …" parent consulting and clinician antibiotic prescribing decisions for children with respiratory tract infections: An analysis across four qualitative studies. *Social Science & Medicine* 2015;136-137:156-64. doi: 10.1016/J.SOCSCIMED.2015.05.027

18. Cheng J, Coope C, Chai J, et al. Knowledge and behaviors in relation to antibiotic use among rural residents in Anhui, China. *Pharmacoepidemiol Drug Saf* 2018;27(6):652-59. doi: 10.1002/pds.4429 [published Online First: 2018/03/28]

19. Currie J, Lin W, Meng J. Addressing Antibiotic Abuse in China: An Experimental Audit Study. *Journal of development economics* 2014;110:39-51. doi: 10.1016/j.jdeveco.2014.05.006

20. Reynolds L, McKee M. Factors influencing antibiotic prescribing in China: An exploratory analysis. *Health Policy* 2009;90:32-36. doi: 10.1016/j.healthpol.2008.09.002

21. Bloom G, Wilkinson A, Tomson G, et al. AMR Addressing Resistance To Antibiotics In Pluralistic Health Systems About the STEPS Centre.

22. Cui D, Liu X, Hawkey P, et al. Use of and microbial resistance to antibiotics in China: a path to reducing antimicrobial resistance. *J Int Med Res* 2017;45(6):1768-78. doi: 10.1177/0300060516686230

23. X T, C Z, YC X, et al. The composition and antimicrobial resistance of isolates from lower respiratory tract and blood in hospitalized patients in respiratory ward: a multicenter

For peer review only

national study in China. *Chinese journal of tuberculosis and respiratory diseases* 2018;41(4):281-87. doi: 10.3760/cma.j.issn.1001-0939.2018.04.007

24. McNulty C, Thomas M, John R, et al. Problems of basing patient recruitment for primary care studies on routine laboratory submissions. *J Clin Pathol* 2007;60(11):1290-3. doi: 10.1136/jcp.2007.046854
25. Chin TL, MacGowan AP, Bowker KE, et al. Prevalence of antibiotic resistance in Escherichia coli isolated from urine samples routinely referred by general practitioners in a large urban centre in south-west England. *J Antimicrob Chemother* 2015;70(7):2167-9. doi: 10.1093/jac/dkv050 [published Online First: 2015/03/15]
26. Chin TL, McNulty C, Beck C, et al. Antimicrobial resistance surveillance in urinary tract infections in primary care: Table 1. *Journal of Antimicrobial Chemotherapy* 2016;71:2723-28. doi: 10.1093/jac/dkw223
27. Qiao LD, Chen S, Yang Y, et al. Characteristics of urinary tract infection pathogens and their in vitro susceptibility to antimicrobial agents in China: data from a multicenter study. *BMJ Open* 2013;3(12):e004152. doi: 10.1136/bmjopen-2013-004152
28. Kim SH, Song JH, Chung DR, et al. Changing trends in antimicrobial resistance and serotypes of Streptococcus pneumoniae isolates in Asian countries: an Asian Network for Surveillance of Resistant Pathogens (ANSORP) study. *Antimicrob Agents Chemother* 2012;56(3):1418-26. doi: 10.1128/AAC.05658-11
29. Brieger WR. Pile sorts as a means of improving the quality of survey data: malaria illness symptoms. *Health education research* 1994;9:257-60.
30. Quintiliani LM, Campbell MK, Haines PS, et al. The Use of the Pile Sort Method in Identifying Groups of Healthful Lifestyle Behaviors among Female Community College Students. *Journal of the American Dietetic Association* 2008;108:1503-07. doi: 10.1016/j.jada.2008.06.428
31. Michie S, van Stralen MM, West R. The behaviour change wheel: A new method for characterising and designing behaviour change interventions. *Implementation Science* 2011;6:42. doi: 10.1186/1748-5908-6-42

For peer review only

BMJ Open

Pathways to optimising antibiotic use in rural Anhui province, China: identifying key determinants in community and clinical settings, a mixed methods study protocol.

Journal:	*BMJ Open*
Manuscript ID	bmjopen-2018-027819.R1
Article Type:	Protocol
Date Submitted by the Author:	17-Jun-2019
Complete List of Authors:	Zhao, Linhai; Anhui Medical University, School of Health Services Management<br>Kwiatkowska, Rachel; Public Health England, Field Service, National Infection Service; University of Bristol Medical School, School of Population Health Sciences, NIHR Health Protection Research Unit in Evaluation of Interventions<br>Chai, Jing; Anhui Medical University, School of Health Service Management<br>Cabral, Christie; University of Bristol Medical School, Centre for Academic Primary Care<br>Chen, Meixuan; University of Bristol Medical School, School of Population Health Sciences<br>Bowker, Karen; North Bristol NHS Trust, Severn Pathology<br>Coope, Caroline; Public Health England, Field Service, National Infection Service; University of Bristol Medical School, School of Population Health Sciences, NIHR Health Protection Research Unit in Evaluation of Interventions<br>Shen, Jilu; First Affiliated Hospital of Anhui Medical University<br>Shen, XingRong; Anhui Medical University, School of Health Services Management<br>Cheng, Jing; Anhui Medical University, School of Health Services Management<br>Feng, Rui; Anhui Medical University, Library Department of Literature Retrieval and Analysis<br>Kadetz, Paul; Drew University; Xi'an Jiaotong-Liverpool University<br>MacGowan, Alasdair; North Bristol NHS Trust, Severn Pathology<br>Oliver, Isabel; University of Bristol School of Population Health Sciences, NIHR Health Protection Research Unit in Evaluation of Interventions; Public Health England, Field Service, National Infection Service<br>Hickman, M; University of Bristol Medical School, School of Population Health Sciences; University of Bristol Medical School, School of Population Health Sciences, NIHR Health Protection Research Unit in Evaluation of Interventions<br>Wang, DeBin; Anhui Medical University, School of Health Services Management<br>Lambert, Helen; University of Bristol Medical School, School of Population Health Sciences
Primary Subject	Global health

Heading:	
Secondary Subject Heading:	Infectious diseases, Health policy, Epidemiology, Public health
Keywords:	Antimicrobial Resistance, Antibiotics, Mixed methods, China, Outpatient, Medical Records

SCHOLARONE™
Manuscripts

Pathways to optimising antibiotic use in rural Anhui province, China: identifying key determinants in community and clinical settings, a mixed methods study protocol.

Linhai Zhao[1]*; Rachel Kwiatkowska[2,3]*; Jing Chai[1]; Christie Cabral[4]; Meixuan Chen[5]; Karen Bowker[6]; Caroline Coope[2,3]; Jilu Shen[7]; XingRong Shen[1]; Jing Cheng[1]; Rui Feng[8]; Paul Kadetz[9-11] ; Alasdair MacGowan[6]; Isabel Oliver[2,3]; Matthew Hickman[2,5]; Debin Wang[1]; Helen Lambert[5]

1. School of Health Service Management, Anhui Medical University, 81 Meishan Road, Hefei, 230032, China.
2. NIHR Health Protection Research Unit (HPRU) in Evaluation of Interventions, Bristol Medical School, Population Health Sciences, University of Bristol, Oakfield House, Oakfield Grove, Bristol, BS8 2BN, UK
3. Field Service, National Infection Service, Public Health England, 3rd floor, 2 Rivergate, Bristol, UK BS1 6EH, UK
4. Centre for Academic Primary Care, Bristol Medical School, University of Bristol, Canynge Hall, 39 Whatley Road Bristol BS8 2PS, UK
5. Department of Population Health Sciences, Bristol Medical School, Bristol Medical School, Canynge Hall, 39 Whatley Road, Bristol BS8 2PS, UK
6. Severn Pathology, North Bristol NHS Trust, Pathology Building, Southmead Hospital, Southmead Road, Westbury-On-Trym, Bristol BS10 5NB, UK
7. First Affiliated Hospital of Anhui Medical University, 218 Jixi Rd, Shushan Qu, Hefei Shi, Anhui Sheng, China, 230022
8. Library of Anhui Medical University, 81 Meishan Road, Hefei, 230032, China
9. Drew University, 36 Madison Ave, Madison, NJ 07940, USA
10. Xi'an Jiaotong Liverpool University, Wuzhong, Suzhou, China, 215123
11. China Centre for Health and Humanity, University College London, Gower Street, WC1E 6BT, UK

*LZ and RK contributed equally to this manuscript.

Correspondence to: Dr R M Kwiatkowska, Field Service, National Infection Service, Public Health England, 3rd floor, 2 Rivergate, Bristol, UK BS1 6EH. Tel. +44 (0)1179 689226 Email rachel.kwiatkowska@phe.gov.uk.

Keywords: Antimicrobial Resistance; Antibiotics; Mixed Methods; China; Medical Records; Outpatients

Word count (excluding abstract): 3784

Abstract

Introduction: This study aims to investigate patterns of antibiotic treatment-seeking, describe

For peer review only - http://bmjopen.bmj.com/site/about/guidelines.xhtml

current levels of and drivers for antibiotic use for common infections (respiratory tract and urinary tract infections), and test the feasibility of determining the prevalence and epidemiology of antimicrobial resistance (AMR) in rural areas of Anhui Province, in order to identify potential interventions to promote antibiotic stewardship and reduce the burden of AMR in China.

Methods and analysis: We will conduct direct observations, structured and semi-structured interviews in retail pharmacies, village clinics and township health centres to investigate treatment-seeking and antibiotic use. Clinical isolates from 1550 sputum, throat swab and urine samples taken from consenting patients at village and township health centers will be analysed to identify bacterial pathogens and ascertain antibiotic susceptibilities. Healthcare records will be surveyed for a sub-sample of those recruited to the study, to assess their completeness and accuracy.

Ethics and dissemination: The full research protocol has been reviewed and approved by the Biomedical Ethics Committee of Anhui Medical University (reference number: 20170271). Participation of patients and doctors are voluntary and written informed consent is sought from all participants. Findings from the study will be disseminated through academic routes including peer-reviewed publications and conference presentations, via tailored research summaries for health professionals, health service managers and policymakers, and through an end of project impact workshop with local and regional stakeholders to identify key messages and priorities for action.

Strengths and limitations of this study:

- This innovative cross-disciplinary study will combine qualitative, microbiological and epidemiological methodologies to investigate drivers of AMR in rural China.
- It will systematically document key drivers of, and patient pathways leading to, antibiotic use and establish the feasibility of microbiological testing and epidemiological monitoring for AMR and antibiotic use at front-line medical settings in rural China.
- The mixed methods approach will provide a comprehensive picture of factors influencing prescribing and sampling practices, so that bias arising from any individual dataset can be accounted for in the analysis and interpretation of results strengthened through triangulation.
- The study area, though selected to be representative of rural populations in China, only includes three rural counties of 105 counties in Anhui province. As such, results may have limited generalisability.
- Nonetheless, the study will identify modifiable influences on current treatment-seeking pathways leading to antibiotic use and determine the feasibility of measuring and monitoring AMR in rural populations; these findings will inform efforts to reduce inappropriate prescribing and improve antimicrobial stewardship.

For peer review only - http://bmjopen.bmj.com/site/about/guidelines.xhtml

Introduction

The problem of drug-resistant infections is widely acknowledged to be of global public health concern, with non-essential use of antibiotics in humans a major contributor.[1] There is substantial evidence that antibiotics are overused in the Chinese health care system.[2-8] A range of possible reasons for unnecessary use of antibiotics have been identified, including 'demand side' issues such as cultural expectations and social norms leading patients to expect antibiotic treatment, and 'supply side' issues such as perverse financial incentives for prescribing antibiotics, systemic pressures on healthcare providers and professional norms which lead to overprescribing as a risk-reduction strategy. Of these, service providers may play a dominant role.

Health professionals in the UK and China often claim that antibiotic overuse is driven by patient demand and link this, in turn, to lack of public understanding of antibiotic effectiveness and low public awareness. [9] Studies in both the UK and China suggest however that 'patient demand' may actually be a misperception on the part of health care providers, or a rationalisation of their own antibiotic prescribing practices.[10-13] A study of self-medication in Hefei City, Anhui province, found an association between higher maternal education, medical insurance cover and non-prescribed use of antibiotics for children's illnesses.[14] A UK study of prescribing in primary care showed doctors' perceptions of patient pressure to be a consistently stronger predictor of prescribing than patients' actual preferences.[15] On the other hand, it is not uncommon for doctors in both the UK and China to prescribe antibiotics 'just in case', due to a desire to reduce medical, legal and reputational risk in the face of clinical uncertainty.[16 17]

A cluster-randomized survey was conducted during the development of this protocol and 2600 rural residents in 12 counties of Anhui Province were asked about self-reported health service-seeking and antibiotic consumption.[18] Almost three quarters (72.7%) of respondents reported that they would do as told if their doctors let them leave an appointment without prescribing any medicine, and only 14.3% had ever asked their doctors to prescribe specific drugs.[19] Results also suggested that knowledge about antibiotics (which pathogens they can be used to treat, and what the potential risks are of using them) was associated with increased purchasing of over-the-counter pharmaceuticals and with re-use of leftover pharmaceuticals from family or friends.

There is evidence from China that health systems, especially remuneration mechanisms, may be important drivers of unnecessary antibiotic use. [20] Patients are used to paying only for medicines they are given and medicine forms an important source of revenue for all care providers. Historically, remuneration mechanisms have provided a perverse financial incentive for health care practitioners to overprescribe antibiotics, particularly at lower levels of the health system. [21] Recent government health care reforms, together with policies such as the Special Antimicrobial Use Rectification program, have been associated with reductions in antibiotic prescribing at county and higher level public hospitals.[3] However, current policies aim to reduce overall use (for example by capping the overall proportion of antibiotic

For peer review only - http://bmjopen.bmj.com/site/about/guidelines.xhtml

prescriptions at hospitals), but do not necessarily ensure equitable access to essential treatment; restricting overall availability of antibiotics may exacerbate health inequities through its negative effects on access to effective treatment among the poor. [22]

Current policies and interventions mainly focus on antibiotic prescribing, but in many settings non-prescribed antibiotics are widely consumed. In China, antibiotics are freely available over-the-counter without prescription, but little is known for either urban or rural settings about what prompts people to seek treatment at different health facilities or the role of the informal sector (including self-medication, retail pharmacies, traditional Chinese medicine practitioners, unqualified bio-medicine practitioners and home care) in the management of infections.

Microbiological facilities are not available at most front-line health care settings. Most research on AMR in China has therefore drawn on patient data collected in urban teaching hospitals. Because patients can choose to attend facilities at any level of the three-tier health system (community clinics, township health centres, or county-level hospitals) and care pathways do not follow a predictable pattern of upwards referral, the extent to which antibiotic prescribing in urban hospitals drives the spread of antibiotic resistance in the wider population is currently unknown. The often asserted high burden of antimicrobial resistance in China may be misleading since current assumptions are based on potential pathogens isolated in selected microbiology laboratories enrolled in the national surveillance system and it is uncertain if this reflects the incidence of resistance in non-hospitalised patients with mild to moderate infection receiving antibiotics, most of whom do not have samples sent to the laboratory.[23 24] UK data indicates that 1:35 patients presenting to their GPs with acute cough have microbiological sampling and perhaps 1:5 with UTI.[25] This data indicates that resistance rates to key antibiotics based on biased laboratory data may be over-emphasised.[26 27]

There is a need for comprehensive and systematic assessment of prescribing and purchasing practices in the context of China's unique health systems and policies, to identify potential targets for interventions to optimise prescribing and consumption. There are also crucial gaps in evidence regarding antibiotic resistance and its determinants in rural communities and health facilities at village and township levels in China and it is important to investigate the possibility of introducing routine monitoring of prevalence and epidemiology of AMR in these settings.

Study aims and objectives

This study aims to investigate levels of antibiotic prescribing and the burden of AMR in rural areas in China and identify key drivers of antibiotic use for common infections in the community, in order to identify potential interventions to promote antibiotic stewardship and reduce the burden of AMR.

The study objectives are summarised in table 1.

For peer review only - http://bmjopen.bmj.com/site/about/guidelines.xhtml

Table 1 Study objectives

1. Assess feasibility of obtaining community-based samples, isolating common pathogens and determining their antibiotic susceptibilities for presentations of Urinary Tract Infections (UTIs) and Respiratory Tract Infections (RTIs);
2. Estimate prevalence of AMR in non-hospitalised patients and test the hypothesis that community prevalence of AMR is lower than that based on analyses of samples from large urban hospitals with a higher proportion of inpatients;
3. Describe patient clinical record systems and assess the reliability of information from electronic patient records for monitoring antimicrobial usage and AMR;
4. Analyse microbiological findings in relation to patient-reported symptoms, clinical diagnoses and antibiotics prescribed in clinical facilities;
5. Investigate what types and sources of health care rural residents of Anhui Province seek for common respiratory tract infections from all forms and sources of health care (including informal, Traditional Chinese Medicine);
6. Understand what drives antibiotic provision (both prescribed and purchased without prescription) and consumption for selected conditions at lower levels of the formal (government-run facilities) and informal (retail pharmacies and any other treatment sources) health care system; and
7. Assess what types of and targets for intervention are likely to be most effective for optimizing antibiotic use.

Methods and analysis

Study design

This is a mixed-method study comprising two main components: a microbiological feasibility study and clinical record review (objectives 1-4), and a qualitative study of antimicrobial prescribing and purchasing practices (objectives 5-7).

Setting

This study is being implemented in three rural residential areas in Anhui Province, including one village clinic, one township health centre and all local retail pharmacies (4-8) within each area. One of the three areas will be used to pilot the study. Anhui Province is located in northeast China and has a population of 68.6 million of whom 57% live in rural areas. Per capita GDP and income in Anhui rank in the middle (14th) among all provinces in the nation. Its social, cultural and economic background is representative of over 80% of the population in China. There are 968 hospitals, 1,941 community & 1,398 township health centres, and 15,288 village clinics in the province (Annual Health Anhui Statistics 2014). As in most other provinces, there is no strict referral system in Anhui and patients may choose from any level and kind of caregiver, though medical insurance systems enact a 10 to 20% decreasing reimbursement ratio for direct medical expenditures incurred at primary care settings upward. In terms of first time medical care, 62.9% is at village or community clinics, 17.3%, at township or community health

For peer review only - http://bmjopen.bmj.com/site/about/guidelines.xhtml

centres and 13.6%, at county level hospitals (Annual Anhui Health Statistics 2014).

Participants

The microbiological sub-study aims to recruit a total of 1,550 patients (1,000 with exacerbation of COPD and 555 with UTI). We estimate that this will yield 200 *E coli* isolates and 100 *Streptococcus pneumoniae* isolates which should provide sufficient power to allow us to estimate key antibiotic susceptibilities (**objective 4**), on the assumption that:

- 40% of UTI samples will yield a pathogen
- 25% of COPD samples will yield a pathogen
- 90% of pathogens isolated from urine will be *E. coli*
- 40% of pathogens isolated from sputum will be *S. pneumoniae*
- In *E.coli*, resistance to nitrofurantoin, fosfomycin and co-amoxiclav will be <5% and resistance to cephalosporins and fluoroquinolones 50-60% [28]
- In *S. pneumoniae*, penicillin resistance will be 12.5% and erythromycin resistance 90% [29]

Inclusion criteria of patients are male or female who are: a) 18 years or older and able to give consent to participate in the microbiological study, exit survey and/or follow-up interviews; b) presenting to the recruitment site for his/her current illness for the first time during the study period; and c) diagnosed by the attending doctor as having one or more of the following: urinary tract infection, exacerbation of chronic obstructive pulmonary disease, upper respiratory tract infection with productive cough, and sore throat. These patients will be selected via “consecutive sampling” in which, when a start date has been determined, the recruitment continues daily (7 days a week) thereafter, between 8am-5pm or 9am-6pm on alternate days, until the target numbers have been reached. All incoming patients to the site village clinics and township health centres who meet the inclusion criteria during any study day are invited to participate. This is a pragmatic approach to sampling since patient record systems do not allow the flexibility to carry out random sampling, and ongoing recruitment will ensure the most efficient use of staff and resources in this setting.
Qualitative study participants will include: a) patients visiting the selected village clinics and township health centers during the study period; b) customers visiting the selected retail pharmacies during the study period; c) doctors within the clinics or health centers for treatment of RTIs/UTIs; d) salespersons working within the selected retail pharmacies.

Data collection

Specimen collection and microbiological testing (objectives 1-2, 4)

The study will collect sputum, throat swab and urine specimens for pathogen identification and susceptibility testing. Sputum will be collected from RTI patients with productive cough (e.g., exacerbation of Chronic Obstructive Pulmonary Disease (COPD); throat swabs from patients with sore throat; and urine from patients with UTIs. Specimen collection will be performed by the attending doctor and placed into a sterilised tube according to a standard operation protocol. The specimen tubes will be put immediately into a refrigerator and preserved at below –4℃ temporarily prior to being shipped to the Central Laboratory of Anhui Medical University

For peer review only - http://bmjopen.bmj.com/site/about/guidelines.xhtml

(AMU). At about 12:00 pm and 5:30 pm each day, the samples in the refrigerator will be transferred to a portable code case filled with ice bags which is then sealed and handed over to a contracted bus company. The company is held responsible for shipping the specimens within a set time limit (within 4 hours).

Upon arrival of any batch of specimens at AMU central lab, a designated technician will perform standard specimen assessment and pre-treatment and then bacteria inoculation, identification, and susceptibility tests. Sputum and throat swab specimens will be inoculated on four plates (a blood agar plate, a MacConkey agar plate, a chocolate agar plate and a chrome agar plate); and urine specimens inoculated on two plates (a blood agar plate and a MacConkey agar plate). The inoculated blood agar and chocolate agar plates will be cultured and incubated in r 5% CO2 and 35^{0}C; the MacConkey agar and the chrome agar plates, incubated in air at 35^{0}C. The incubation time will be 18-24 hours plus an additional 24 hours if no bacteria are observed for the first period. Bacteria identification will use automated methods i.e., the MicroscanWalkaway-96 System with PC33 (for gram positive bacteria) or NC50 (for gram negative bacteria) kits, and also according to different biochemical reactions as follows: a) E.coli chrome agar/ VITEK2 or MALDI-TOF; b) S.pneumoniae optochin/ biochemistry; c) H.influenzae X+V factors or MALDI-TOF; and d) M.catarrhalis MALDI-TOF. For the bacteria susceptibilities, automated methods will be used (VITEK2 and MicroScan Walk Away 96 plus) along with disk diffusion and agar dilution methods (according to CLSI). Multi-drug resistance will be defined according to standard criteria as set out by Magiorakos et al.[30]Antimicrobial susceptibility data on the key pathogens identified in community sampling will be extracted from the existing diagnostic laboratory databases for comparison with isolates from village clinics and county hospitals. A timeline for data collection can be found in Table 1 of the appendix; a full list of bacterial isolates and susceptibility testing can be found in Table 2 of the appendix.

Electronic Record Review (objectives 3, 4)

Medical record-keeping systems in Anhui province will be described in detail using information from clinic observations and consultations with clinical personnel and medical centre directors. Outpatient electronic care systems will be interrogated two weeks or more after patient recruitment and compared with notes from clinic observations to assess completeness and accuracy of electronic care records. A one year sample of all electronic care records from the participating facilities will then be drawn to investigate patterns and rates of antibiotic prescribing in relation to recorded diagnosis, clinical indication and laboratory results.

Structured exit surveys (objectives 5, 6)

Exit surveys will be carried out with patients presenting with symptoms of sore throat, respiratory tract infection (RTI) or urinary tract infection (UTI) following either their consultation at a health care facility or their visit to a retail pharmacy. Those who consent to being recruited will complete the survey prior to leaving the facility. Using a brief semi-structured questionnaire developed from open-ended interview responses in the study's pilot

For peer review only - http://bmjopen.bmj.com/site/about/guidelines.xhtml

phase, the survey will solicit details of symptoms, perception of the problem (lay diagnosis), professional and/or self-treatment taken before the visit, whether the diagnosis and treatment received (including prescribed antibiotics) is as expected, as well as views on specimen collection and microbiological testing in patients attending the health care facility.

Semi-structured observations (objectives 3, 5, 6)

These will occur at all the participating clinics, health centers and retail pharmacies. At clinics and health centers, the observation focuses on daily operational routine including test ordering, prescribing, patient recall and other standard procedures using a pre-designed worksheet, together with observational notes on clinical encounters. At retail pharmacies, the observation uses a similar worksheet and documents daily encounters between customers and shop assistants including the health problems presented, and medicines and information asked for by the customers and/or suggestions made, and medicines and information given by the assistants.

In-depth interviews (objectives 5, 6)

These will involve a sub-sample of the patients (previous experience of similar studies suggests that around 60 in total are likely to be needed to reach data saturation) and customers (around 60) who have completed the exit survey and all the related doctors and salespersons who have served the participating patients or costumers. Interviews with individual patients and customers will be undertaken respectively at their homes and at the pharmacy one to two weeks after their initial visit; interviews with individual doctors and salespersons will take place any time during the study period when they are free. Topic guides refined during the pilot phase of the study will be used to focus patient interviews on the illness episode for which treatment was sought and any previous action taken to manage the illness, while interviews with doctors focus on the following main topics: professional experience and expertise, normal practice, prescribing antibiotics, patient influences on antibiotic prescribing and external influences on antibiotic prescribing.

A sub-sample of interviewees will take part in pile sorts, a qualitative method in which informants are provided with a set of index cards each showing a pertinent term and asked to group the cards according to their perceived appropriate clustering of terms. Terms will be antibiotics-related and derived from earlier exit surveys, observations and in-depth interviews.[31 32] This method provides another means to distinguish how informants understand antibiotics and their use.

Data analysis

Quantitative analysis (objectives 1-4)

Data from the microbiological testing, review of clinical records and exit surveys will be analysed quantitatively to investigate prevalence of infection and rates of resistance, relationships between infection/ resistance rates and patient characteristics, and feasibility of routine microbiological testing in rural clinics. The feasibility of obtaining community-based samples of common pathogens and determining their antibiotic sensitivities **(objective 1)** will

For peer review only - http://bmjopen.bmj.com/site/about/guidelines.xhtml

be examined by: a) ratios of patients who have provided specimens versus those who have refused and their reasons for doing so; b) percentages of specimens assessed as being properly collected, preserved and shipped by designated microbiological technicians; c) ratios of positive versus negative attitudes toward giving specimens among the exit interviewees and views of the participating doctors. Prevalence of pathogens and antimicrobial resistance **(objective 2)** will be assessed by: a) estimating percentages of pathogen positive specimens in terms of all and specific bacteria by diagnosis, type of specimen, patient reported symptoms, previous treatment history, and socio-demographics; b) calculating percentages of resistant pathogens by type of bacteria and antibiotics; and c) comparing, using chi-square-test, these pathogen and resistance rates in non-hospitalised patients with those from analyses of data from large urban hospitals. We will produce a descriptive analysis of patient record systems and electronic record completeness and accuracy **(objective 3)**. If sample sizes allow it, descriptive analyses may be followed by analytical studies to explore relationships between pathogen types and susceptibilities and patient characteristics such as presenting symptoms, clinical diagnosis, prior use of antibiotics and demographics **(objective 4).**

Qualitative analysis (objectives 5-7)

Analysis of the qualitative data (in-depth interviews, observations) will focus on identifying characteristic patterns in treatment-seeking pathways to antibiotic use among patients and antibiotic purchasers (**objective 5**) and on identifying key social, cultural, health systems and behavioural determinants of both antibiotic prescribing and provision among health care providers and of antibiotic use among residents (**objective 6**). Thematic and framework analysis will be undertaken for each of the four qualitative datasets using a similar approach. Following translation of a subset of interviews from each data set, members of the research team will independently free code a minimum of three interview transcripts in Mandarin or English and then meet to compare and finalise a code list. Further interviews will be coded thematically in batches in the software package NVivo 12 using the agreed codes until all data have been coded. Each interviewer will also summarise each interview transcript by entering a summary of the coded data in a Framework using Excel. Pile sorts will be analysed using Johnson's Hierarchical Cluster analysis, providing a two-dimensional concept mapping of the terms that are most frequently grouped together.

The occurrence of resulting themes will also be used to produce theme frequency maps and theme networks via NVivo or UCINET for different groups of participants and different topics.

Analysis of antibiotics-related pathways comprises identifying both a complete set of paths for common RTIs/UTIs and factors affecting patient trajectories among these pathways. Anticipated path sets to be produced include a general path set applicable for both RTIs and UTIs and an RTI and UTI-specific path set respectively. Similarly, factor sets of interest in this study comprise combinations of factors that affect selection between: a) doing nothing versus taking action; b) self-care versus professional treatment; c) western medicine versus traditional Chinese medicine; d) antibiotics versus non-antibiotics therapy.

For peer review only - http://bmjopen.bmj.com/site/about/guidelines.xhtml

Analytic synthesis

Development of interventions for optimizing antibiotics use (**objective 7**) will build upon findings from both quantitative and qualitative analyses. It will also analyse the pathways and determinants identified from this study in relation to the Behaviour Change Wheel, a theoretical framework developed from 19 frameworks of behaviour change identified in a systematic literature review.[33] The COM-B ('capability', 'opportunity', 'motivation' and 'behaviour') model, which recognizes behaviour as part of an interacting system, will be used to identify key modifiable influences leading to the use of antibiotics and subsequently, inform recommendations for interventions to optimise antibiotic use.

Patient and Public Involvement

Development of the research question was informed by patients' experiences and preferences as documented in a previous study.[19] The results will be disseminated to study participants and other stakeholders through a local dissemination workshop to be held at the end of this study and through short summaries of research findings.

Ethics and dissemination

This protocol is rooted in a multidisciplinary collaboration between institutions in the UK and China. Investigators will not only benefit from exchange of academic knowledge and methodologies but will gain a situational awareness which extends beyond their own area of expertise. This will lead to greater depth of insight into the scope and interrelatedness of influencing factors and allow the team to analyse and interpret study results with full awareness of the complexity of drivers of AMR and antibiotic use in rural Anhui province. The research will provide academics with a documented case study of how to conduct large scale research involving a multi-disciplinary team across two countries, as well as a ‘worked example’ of research capacity-building through academic collaboration for the benefit of a middle-income country. Regular workshops will allow the China and UK research teams to exchange ideas and insights throughout the duration of the study. Lessons from this study will be shared with the wider academic community through peer-reviewed publications and conference presentations.

This innovative study will provide the first systematic documentation of routes and means to antibiotic use by residents at lower ends of the healthcare system (township and village clinics) in China and assess the feasibility of routine microbiological testing and analysis of antimicrobial sensitivities in the context of information from clinic healthcare records.

Findings from the study will be summarised for the benefit of public health policymakers, health professionals and health service managers in China and elsewhere. A final workshop will be held for the purpose of presenting the evidence generated by this study, and working with local and regional stakeholders to translate this evidence into public health action.

For peer review only - http://bmjopen.bmj.com/site/about/guidelines.xhtml

The Biomedical Ethics Committee of Anhui Medical University has provided full ethical approval for this study (reference number: 20170271). Participation in the study is fully voluntary for patients, doctors and pharmacy staff, all of whom will give written informed consent to participate. **Authors' contributions:**

RK, LZ and JChai wrote the manuscript with supervision and contributions from project leads AMacG (microbiology component), IO and MH (record review component), PK (social sciences component) and Principal Investigators HL (UK) and DW (China).

CCo and PK contributed to developing the initial study collaboration and to revision of the manuscript. RF, JCheng, JChai, MC and CCa contributed to qualitative research tool design including methods for data collection and analysis. KB and JS developed methods for microbiology sample collection and laboratory analysis. IO, RK and XS developed methods for record review data extraction and analysis. XS also designed a central database for storing data from all components of the study. HL, AMacG, IO, MH, PK and DW conceived and designed the original study. All authors reviewed the manuscript.

Acknowledgements

The authors gratefully acknowledge the contributions of Professor Susan Michie and Dr. Annegret Schneider (University College London), Professor Alastair Hay (University of Bristol), and Professor Anthony Kessel (Public Health England) in the conception of this study.

Competing interests statement: None

Funding statement: This work was supported by the Newton Fund (UK Research and Innovation (UKRI) and the National Natural Science Foundation of China (NSFC)) under the UK-China AMR Partnership Initiative, grant number MR/P00756/1.

RK, CCo, MH and IO all acknowledge support from the NIHR Health Protection Research Unit in Evaluation of Interventions at the University of Bristol.

References

1. WHO. Global Action Plan on Antimicrobial Resistance: World Health Organisation, 2015.
2. Li Y, Xu J, Wang F, et al. Overprescribing In China, Driven By Financial Incentives, Results In Very High Use Of Antibiotics, Injections, And Corticosteroids. *Health Affairs* 2012;31:1075-82. doi: 10.1377/hlthaff.2010.0965
3. Sun Q, Dyar OJ, Zhao L, et al. Overuse of antibiotics for the common cold – attitudes and behaviors among doctors in rural areas of Shandong Province, China. *BMC Pharmacology and Toxicology* 2015;16:6. doi: 10.1186/s40360-015-0009-x
4. Xiao Y, Li L. Legislation of clinical antibiotic use in China. *The Lancet Infectious Diseases* 2013;13(3):189-91. doi: 10.1016/s1473-3099(13)70011-2

For peer review only - http://bmjopen.bmj.com/site/about/guidelines.xhtml

5. Xiao Y, Zhao K, Bishai DM, et al. Essential drugs policy in three rural counties in China: what does a complexity lens add? *Soc Sci Med* 2013;93:220-8. doi: 10.1016/j.socscimed.2012.09.034 [published Online First: 2012/10/30]

6. Yang C, Yang S, Yang L, et al. The impact of the national essential medicines policy on rational use of antibiotics in China. *Chinese Journal of Health Service Management* 2014;308:109-12.

7. Yin X, Song F, Gong Y, et al. A systematic review of antibiotic utilization in China. *Journal of Antimicrobial Chemotherapy* 2013;68:2445-52. doi: 10.1093/jac/dkt223

8. Zhang R, Eggleston K, Rotimi V, et al. Antibiotic resistance as a global threat: evidence from China, Kuwait and the United States. *Global Health* 2006;2:6. doi: 10.1186/1744-8603-2-6 [published Online First: 2006/04/11]

9. Cole A. GPs feel pressurised to prescribe unnecessary antibiotics, survey finds. *BMJ (Clinical research ed)* 2014;349:g5238. doi: 10.1136/BMJ.G5238

10. Brookes-Howell L, Wood F, Verheij T, et al. Trust, openness and continuity of care influence acceptance of antibiotics for children with respiratory tract infections: a four country qualitative study. *Family Practice* 2014;31:102-10. doi: 10.1093/fampra/cmt052

11. Cabral C, Horwood J, Hay AD, et al. How communication affects prescription decisions in consultations for acute illness in children: a systematic review and meta-ethnography. *BMC Family Practice* 2014;15:63. doi: 10.1186/1471-2296-15-63

12. Lucas PJ, Cabral C, Hay AD, et al. A systematic review of parent and clinician views and perceptions that influence prescribing decisions in relation to acute childhood infections in primary care. *Scandinavian Journal of Primary Health Care* 2015;33:11-20. doi: 10.3109/02813432.2015.1001942

13. McCullough AR, Rathbone J, Parekh S, et al. Not in my backyard: a systematic review of clinicians' knowledge and beliefs about antibiotic resistance. *J Antimicrob Chemother* 2015;70(9):2465-73. doi: 10.1093/jac/dkv164 [published Online First: 2015/06/21]

14. Bi P, Tong S, Parton KA. Family self-medication and antibiotics abuse for children and juveniles in a Chinese city. *Social science & medicine (1982)* 2000;50:1445-50.

15. Little P, Dorward M, Warner G, et al. Importance of patient pressure and perceived pressure and perceived medical need for investigations, referral, and prescribing in primary care: nested observational study. *BMJ* 2004;328:444-0. doi: 10.1136/bmj.38013.644086.7C

16. He AJ. The doctor–patient relationship, defensive medicine and overprescription in Chinese public hospitals: Evidence from a cross-sectional survey in Shenzhen city. *Social Science & Medicine* 2014;123:64-71. doi: https://doi.org/10.1016/j.socscimed.2014.10.055

17. Cabral C, Lucas PJ, Ingram J, et al. “It's safer to …” parent consulting and clinician antibiotic prescribing decisions for children with respiratory tract infections: An analysis across four qualitative studies. *Social Science & Medicine* 2015;136-137:156-64. doi: 10.1016/J.SOCSCIMED.2015.05.027

18. Chai J CC, Cheng J, Oliver I, Kessel A, Hu Z, Wang D. Cross-sectional study of the use of antimicrobials following common infections by rural residents in Anhui, China. *BMJ Open* 2019;9 doi: 10.1136/ bmjopen-2018-024856

19. Cheng J, Coope C, Chai J, et al. Knowledge and behaviors in relation to antibiotic use among rural residents in Anhui, China. *Pharmacoepidemiol Drug Saf* 2018;27(6):652-59. doi: 10.1002/pds.4429 [published Online First: 2018/03/28]

For peer review only - http://bmjopen.bmj.com/site/about/guidelines.xhtml

20. Currie J, Lin W, Meng J. Addressing Antibiotic Abuse in China: An Experimental Audit Study. *Journal of development economics* 2014;110:39-51. doi: 10.1016/j.jdeveco.2014.05.006
21. Reynolds L, McKee M. Factors influencing antibiotic prescribing in China: An exploratory analysis. *Health Policy* 2009;90:32-36. doi: 10.1016/j.healthpol.2008.09.002
22. Bloom G, Wilkinson A, Tomson G, et al. AMR Addressing Resistance To Antibiotics In Pluralistic Health Systems About the STEPS Centre.
23. Cui D, Liu X, Hawkey P, et al. Use of and microbial resistance to antibiotics in China: a path to reducing antimicrobial resistance. *J Int Med Res* 2017;45(6):1768-78. doi: 10.1177/0300060516686230
24. X T, C Z, YC X, et al. The composition and antimicrobial resistance of isolates from lower respiratory tract and blood in hospitalized patients in respiratory ward: a multicenter national study in China. *Chinese journal of tuberculosis and respiratory diseases* 2018;41(4):281-87. doi: 10.3760/cma.j.issn.1001-0939.2018.04.007
25. McNulty C, Thomas M, John R, et al. Problems of basing patient recruitment for primary care studies on routine laboratory submissions. *J Clin Pathol* 2007;60(11):1290-3. doi: 10.1136/jcp.2007.046854
26. Chin TL, MacGowan AP, Bowker KE, et al. Prevalence of antibiotic resistance in Escherichia coli isolated from urine samples routinely referred by general practitioners in a large urban centre in south-west England. *J Antimicrob Chemother* 2015;70(7):2167-9. doi: 10.1093/jac/dkv050 [published Online First: 2015/03/15]
27. Chin TL, McNulty C, Beck C, et al. Antimicrobial resistance surveillance in urinary tract infections in primary care: Table 1. *Journal of Antimicrobial Chemotherapy* 2016;71:2723-28. doi: 10.1093/jac/dkw223
28. Qiao LD, Chen S, Yang Y, et al. Characteristics of urinary tract infection pathogens and their in vitro susceptibility to antimicrobial agents in China: data from a multicenter study. *BMJ Open* 2013;3(12):e004152. doi: 10.1136/bmjopen-2013-004152
29. Kim SH, Song JH, Chung DR, et al. Changing trends in antimicrobial resistance and serotypes of Streptococcus pneumoniae isolates in Asian countries: an Asian Network for Surveillance of Resistant Pathogens (ANSORP) study. *Antimicrob Agents Chemother* 2012;56(3):1418-26. doi: 10.1128/AAC.05658-11
30. Magiorakos AP, Srinivasan A, Carey RB, et al. Multidrug-resistant, extensively drug-resistant and pandrug-resistant bacteria: an international expert proposal for interim standard definitions for acquired resistance. *Clin Microbiol Infect* 2012;18(3):268-81. doi: 10.1111/j.1469-0691.2011.03570.x
31. Brieger WR. Pile sorts as a means of improving the quality of survey data: malaria illness symptoms. *Health education research* 1994;9:257-60.
32. Quintiliani LM, Campbell MK, Haines PS, et al. The Use of the Pile Sort Method in Identifying Groups of Healthful Lifestyle Behaviors among Female Community College Students. *Journal of the American Dietetic Association* 2008;108:1503-07. doi: 10.1016/j.jada.2008.06.428
33. Michie S, van Stralen MM, West R. The behaviour change wheel: A new method for characterising and designing behaviour change interventions. *Implementation Science* 2011;6:42. doi: 10.1186/1748-5908-6-42

For peer review only - http://bmjopen.bmj.com/site/about/guidelines.xhtml

Appendix

Study dates: June 2016 to November 2019

Table 1 Data collection

Component	Start Date Pilot	End Date Pilot	Start Date Site 1	End Date Site 1	Start Date Site 2	End Date Site 2	Start Date Site 3	End Date Site 3
Microbiology	Month 11	Month 12	Month 16	Month 20	Month 21	Month 24	Month 28	Month 31
Exit Interviews	Month 11	Month 12	Month 16	Month 20	Month 21	Month 24	Month 28	Month 31
Patient In-Depth Interviews	Month 12	Month 13	Month 19	Month 20	Not required	Not required	Not required	Not required
Recruitment Proforma [Observations]	Month 10	Month 12	Month 16	Month 20	Month 21	Month 24	Month 28	Month 31
Pharmacy Observations & Exit Interviews	Month 12	Month 13	Month 19	Month 20	Month 23	Month 24	Not required	Not required
Record Review	Month 13	Ongoing review	Month 25	Ongoing review	Month 25	Ongoing review	Not required	Not required

For peer review only

Table 2 Organisms to be analysed related to specimen and antibiotics tested

specimen	isolate	agents tested
urine	*E.coli*<br>*Klebsiella spp*<br>*Proteus spp*	amoxicillin, co-amoxiclav, trimethoprim/ co-trimoxazole, ciprofloxacin, gentamicin, cefotaxime/ceftriaxone
throat swabs	*B.haemolytic streptococci*<br>*Haemophilus spp*	penicillin, macrolide, Blactamase production, quinolone
	Moraxella catarrhalis<br>*S.aureus*	Blactamase production, cefoxitin/oxacillin, macrolide
	S.pneumoniae<br>*Enterobacteriales*<br>*N.meningitidis*	penicillin, macrolide, amoxicillin, co-amoxiclav, trimethoprim/ co-trimoxazole, ciprofloxacin, gentamicin, cefotaxime/ceftriaxone
sputum	*S.pneumoniae*<br>*Haemophilus spp*<br>*Moraxella catarrhalis*<br>*S.aureus*<br>*Enterobacteriales*<br>*B.haemolytic streps*<br>*N.meningitidis*	agents to test as for throat swabs

For peer review only
